# Supplementary material for: Mechanisms governing the pioneering and redistribution capabilities of the non-classical pioneer PU.1
Source: Nat Commun. 2020 Jan 21;11:402. doi: 10.1038/s41467-019-13960-2 (PMC6972792; doi:10.1038/s41467-019-13960-2)
Supplement: Supplementary file 7 — Source data [file 41467_2019_13960_MOESM7_ESM.zip › Source_Data/FigureS2/FigureS2B_MotifScanOutput/KmeansCluster3/homerResults/motif4.similar.html]

motif4

## Information for motif4

T
G
C
A
C
G
T
A
T
G
C
A
T
C
A
G
T
C
A
G
A
T
C
G
C
T
G
A
C
G
T
A
A
T
G
C
  
Reverse Opposite:  

A
T
C
G
A
C
G
T
A
G
C
T
T
A
G
C
A
G
T
C
A
G
T
C
A
C
G
T
G
C
A
T
A
C
G
T
  

|  |  |
| --- | --- |
| p-value: | 1e-336 |
| log p-value: | -7.738e+02 |
| Information Content per bp: | 1.682 |
| Number of Target Sequences with motif | 1910.0 |
| Percentage of Target Sequences with motif | 40.05% |
| Number of Background Sequences with motif | 6330.8 |
| Percentage of Background Sequences with motif | 14.93% |
| Average Position of motif in Targets | 99.3 +/- 51.3bp |
| Average Position of motif in Background | 100.6 +/- 73.4bp |
| Strand Bias (log2 ratio + to - strand density) | -0.2 |
| Multiplicity (# of sites on avg that occur together) | 1.12 |
| Motif File: | file (matrix) reverse opposite |

### Similar de novo motifs found

|  |  |  |  |  |  |  |  |
| --- | --- | --- | --- | --- | --- | --- | --- |
| Rank | Match Score | Redundant Motif | P-value | log P-value | % of Targets | % of Background | Motif file |
| 1 | 0.772 | G C T A C G T A C T G A T C A G C A T G A C T G C T A G C G T A | 1e-145 | -335.228433 | 26.53% | 11.88% | motif file (matrix) |
| 2 | 0.764 | A C T G A C T G A T G C A C G T A C G T A G T C G A T C G A T C A G C T A G C T A C G T A G C T A G C T | 1e-62 | -143.885906 | 3.96% | 0.71% | motif file (matrix) |
| 3 | 0.771 | C T G A T C A G T G C A C G T A T C G A C G T A C T A G C T A G A C T G G T C A G T C A A T G C | 1e-57 | -131.865790 | 2.39% | 0.22% | motif file (matrix) |
